# Challenges and opportunities of LGBTQ health nudging

**DOI:** 10.1002/hsr2.1811

**Published:** 2024-01-08

**Authors:** Yudai Kaneda, Mira Namba

**Affiliations:** ^1^ School of Medicine Hokkaido University Hokkaido Japan; ^2^ School of Medicine Keio University Tokyo Japan

In modern healthcare communication, “nudges”—tools from behavioral economics—have become influential in promoting positive health behaviors.^1^ The lesbian, gay, bisexual, transgender, and queer/questioning (LGBTQ) community, accounting for 8%–13% of the Japanese population, with its diverse health concerns, could benefit from nudges, given research highlighting increased health risks of LGBTQ community such as cancer, mental disorders, and sexually transmitted diseases.^2^, ^3^ Specifically for example, while the annual incidence rate of anal cancer in men is 1.5 cases per 100,000 person‐years, it is significantly higher among HIV‐positive men who have sex with men (MSM) at 45.9 per 100,000 person‐years and 5.1 per 100,000 person‐years among HIV‐negative MSM, with the latter still exceeding the rate observed in the general population; similarly, in women, heterosexual women have a significantly lower prevalence of cervical cancer (14%) compared to lesbian (16.5%) and bisexual women (41.2%).^3^ However, although Japan has universal healthcare insurance, barriers that are deeply rooted in social determinants of health, like stigma, lack of culturally competent care, and limited medical service access, contribute to a significant gap in awareness and regular screenings.^2^, ^4^ This results in many sexual and gender minorities not seeking medical assistance due to fear of incomprehension, discrimination, and rejection by healthcare providers.^2^ Thus, it is imperative for the medical community to thoroughly assess and understand these concerns, especially when considering gender‐specific screenings. Moreover, the challenge lies in increasing screening rates without unintentionally violating an individual's privacy.

An incident in Japan involving a transgender man who, while living as a male, is legally recognized as female, reported in January 2023, highlights a pressing concern.^5^ A corporate employee, a transgender man, residing in Tokyo's Adachi ward, expressed distress upon receiving a postcard notifying him of a screening for “female‐specific cancers.” The postcard's exterior displayed content that inadvertently revealed his legal gender as female to any onlooker. Living in a shared house where mailboxes are communal, there was potential for involuntary outings—a serious concern. To worsen the situation, he received another notification about a “breast cancer prevention set.” Though he was the first to access the mailbox on both occasions, the risk of exposure remains an alarming possibility.

Adachi ward's resident registration system sends notifications based on registered gender and age. While it is crucial to promote early detection of cancers such as breast and cervical, which have higher incidence rates among younger demographics, there is a contentious issue regarding this particular individual. Despite previously requesting the ward to abstain from sending gender‐specific notifications, an administrative oversight resulted in his receipt of a breast cancer prevention kit. The postcard openly revealed his transgender status, risking unintended disclosure to his housemates. Such inadvertent revelations can lead to both personal and societal implications, potentially placing individuals in uncomfortable, if not perilous, situations. Furthermore, it is plausible to assume that some transgender individuals may not have reported their status to official agencies. Such unintended recognition carries risks, emphasizing the need to protect their rights and privacy.

The method adopted by the Adachi ward, wherein the envelope detailed both the content and the deadline, epitomizes a strategy rooted in the Easy, Attractive, Social, and Timely (EAST) framework of nudging to amplify participation rates in screenings. The overarching intent behind this was to channel individuals towards healthier decision‐making paradigms, such as routine medical screenings. The burgeoning global momentum surrounding nudging in public health initiatives bespeaks its efficacy. However, the deployment of such nudges, particularly in sensitive domains like medical communication, mandates rigorous scrutiny. While promoting health consciousness and early detection is commendable, this case highlights the risks, especially concerning inadvertent privacy breaches. Nevertheless, ensuring that these strategies resonate with foundational values of respect, confidentiality, and individual rights becomes imperative in their pragmatic implementation.

Considering these issues, a comprehensive communication approach is essential. The middle ground of advancing health screenings through nudging and preserving individual rights might lie in modern technological advancements and a deeper understanding of the communities. Leveraging online technologies for personalized notifications is a potential solution. From the perspective of the availability heuristic, which suggests that humans often base their decisions on what they frequently see, health agencies could use encrypted email reminders or secured portals to both nudge individuals towards screenings and preserve privacy.^6^ Such methods would also permit a more tailored approach, with content tailored to an individual's specific health needs and gender identity. Furthermore, to amplify the reach and effectiveness of these nudges, health departments could consider partnerships with apps or platforms frequently used by transgender individuals.^7^ This would ensure messages reach the intended audience and foster trust by presenting information in spaces where the transgender community already feels safe and represented. Another possibility is to solicit feedback directly from the LGBTQ community when designing these notifications. Involving them in the crafting process ensures culturally competent and respectful communications. This balanced approach would promote health screenings while respecting individual privacy.

Of note, it is important to acknowledge that while nudges are effective in guiding immediate behavioral decisions, there is a paucity of comprehensive understanding regarding their long‐term impacts.^8^ Moreover, globally, organizations such as GLMA and the Center of Excellence on LGBTQ+ Behavioral Health Equity play a pivotal role in fostering partnerships, providing resources, expertise, and engaging in collaborative advocacy to promote LGBTQ health equity; however, such initiatives are significantly underdeveloped in Japan. Given Japan's classification as a nudge‐cautious country,^9^ indicating a potential reluctance towards embracing nudges, it becomes imperative to strive towards the establishment of a society that fully includes and supports the LGBTQ community, by simultaneously undertaking health education initiatives that,^10^ while more time‐consuming, are anticipated to have a long‐term impact in fostering an inclusive environment for LGBTQ individuals.

In conclusion, while nudges provide valuable insights for public health, its application in areas like healthcare and within communities like the LGBTQ demands great sensitivity. In this context, the incident in the Adachi ward underscores the importance of striking a balance between advancing health screenings and preserving individual rights. As we harness modern technologies to communicate more effectively, our strategies must evolve to be both informative and respectful, keeping in line with the broader societal move towards inclusivity and understanding.

## AUTHOR CONTRIBUTIONS

**Yudai Kaneda**: Conceptualization; data curation; writing—original draft. **Mira Namba**: Writing—review and editing.

## CONFLICT OF INTEREST STATEMENT

The authors declare no conflicts of interest.

## Data Availability

Not applicable.
